# A Synchronous Triggering Method for Impact Artificial Seismic Source and Seismographs Based on Non-Contact Audio Detection

**DOI:** 10.3390/s26082413

**Published:** 2026-04-15

**Authors:** Wei Wang, Yukaichen Yang, Shihe Wang, Zizhuo Wang, Jun Hu, Yongheng Shi, Zhihong Fu

**Affiliations:** 1 School of Electrical Engineering, Chongqing University, Chongqing 401331, China; cqu_ww@126.com (W.W.); 20230889@stu.cqu.edu.cn (S.W.); wangzizhuo@stu.cqu.edu.cn (Z.W.); 20232951@stu.cqu.edu.cn (J.H.); 20230882@stu.cqu.edu.cn (Y.S.); fuzhihong@cqu.edu.cn (Z.F.); 2 School of Electrical Engineering, Xi’an Jiaotong University, Xi’an 710049, China

**Keywords:** artificial seismic source, synchronous triggering, sound recognition, MFCC

## Abstract

Impact artificial seismic sources are gaining popularity in reflection seismic exploration. However, challenges arise due to the uncertain delay between the hammer’s acceleration and its impact on the interface, as well as the strong vibrations or pulsed magnetic fields produced during the acceleration process. These factors complicate the synchronous triggering methods typically used in traditional explosive and sledgehammer artificial seismic sources, often resulting in temporal misalignment of the acquired data. To tackle this issue, this study introduces a high-precision synchronous triggering method based on non-contact audio detection. Utilizing an STM32F4 microcontroller as the core hardware, the system collects ambient audio and extracts 39-dimensional acoustic features via Mel-frequency cepstrum coefficients (MFCC). A lightweight convolutional neural network (CNN) model is employed to accurately identify hammer impact events. Additionally, a synchronization time compensation mechanism is implemented to address system processing delays. Results from 300 field tests conducted in three environments—open ground, construction site, and mining tunnel—demonstrate that the system achieves a triggering accuracy of up to 94.6%, with compensated triggering time errors controlled within ±125 μs, thereby meeting the minimum tolerable synchronous triggering error requirement. This study significantly enhances the reliability of impact-type Artificial Seismic Source exploration data and offers insights for the application of sound recognition in engineering surveying and other related fields.

## 1. Introduction

Reflection seismic exploration is a widely utilized geological exploration technique that employs artificially generated seismic waves to capture reflection and refraction signals from subsurface materials, allowing for the investigation of underground geological structures [1]. In terms of instrumentation, it is essential to use a high-performance artificial seismic source and seismic acquisition tools [2]. Moreover, the artificial seismic source must provide the seismic acquisition instrument with a triggering signal that is perfectly synchronized with the timing of its generated seismic wave. This synchronization is critical for maintaining the temporal consistency of the seismic wave data and significantly influences the overall results of the exploration [3,4].

Traditionally, explosive seismic sources have been relied upon to generate these seismic waves. However, due to concerns regarding safety, environmental protection, and usability constraints, the use of sledgehammer artificial seismic sources has been on the decline, mainly due to their poor energy efficiency and inconsistent performance. In recent years, research and application of impact-type artificial seismic sources [5], such as electromagnetically driven or high-pressure gas-driven artificial seismic sources, have gained considerable attention [6].

**Figure 1:**
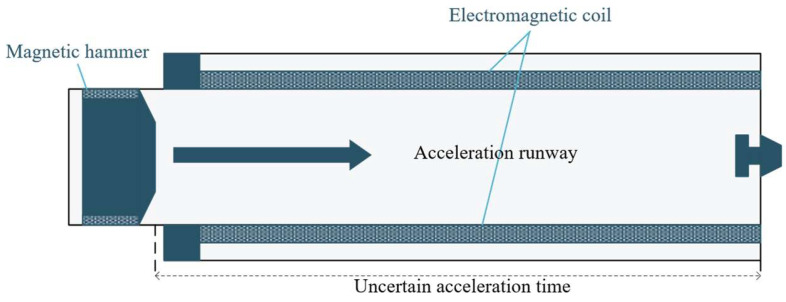
Working principle of the electromagnetically driven artificial seismic source.

However, a significant challenge arises in the practical application of artificial seismic sources: there is an acceleration phase of variable duration between the hammer’s start-up acceleration and the release of impact energy. As illustrated in Figure 1, which presents the working principle of the electromagnetically driven impact seismic source, it is evident that while the start-up acceleration time of the hammer is known, the duration of the hammer’s acceleration remains uncertain. This uncertainty complicates the accurate calculation of the actual impact time based solely on the start-up acceleration time [7]. Utilizing the hammer’s acceleration start signal as the trigger time can introduce considerable time deviations, often exceeding the minimum tolerable synchronous triggering error of 150 μs, resulting in the loss of 3 to 4 samples. Such misalignment in the timing of seismic records can significantly degrade data quality and interpretation. Therefore, investigating a high-precision synchronous triggering method tailored for artificial seismic sources is crucial for enhancing the reliability and effectiveness of exploration data.

In the realm of synchronous triggering technology between artificial seismic sources and seismic acquisition instruments, numerous scholars have conducted comprehensive research on explosive seismic sources. For example, Wen Hengcong designed a triggering system utilizing a vibration circuit for channel wave seismic exploration, which replaced the conventional pulse triggering mechanism [8]. Xu Jingmao proposed an innovative approach involving a falling-edge internal triggering circuit, with the trigger coil wound around the detonator, thereby effectively addressing the limitations associated with external triggering and rising-edge triggering [9]. Additionally, Li Wenqiang developed a system that harnesses the high temperature produced during an explosion to melt the insulation layer of enameled wire, generating a falling edge triggering signal to satisfy the synchronization requirements of multiple devices in explosion experiments [10]. However, since artificial seismic sources lack a detonator structure and do not produce explosive chemical reactions, the aforementioned methods cannot be directly applied. Thus, there is a pressing need to develop a specialized synchronous triggering scheme tailored for this specific type of seismic source.

The team has systematically investigated various synchronous triggering schemes in previous research. The traditional broken wire triggering method used in explosive sources has demonstrated insufficient reliability, primarily due to the buffering characteristics of the hammer impact surface and the conductivity properties of the hammer body [11]. Since most media at the impacted interface exhibit buffering properties, such broken wire short circuit triggering is prone to significant lag or complete failure to trigger. Additionally, piezoelectric vibration sensors struggle to operate reliably due to strong mechanical vibration interference [12]. Non-contact solutions, such as photoelectric switches, encounter challenges from the strong magnetic interference generated by the vibration source itself, as well as limited installation conditions [13]. Overall, these solutions have not yielded ideal results, largely due to the unpredictability of the impacted medium and the vulnerability of the synchronous triggering device to interference.

In recent years, sound recognition technology has emerged as a promising non-contact sensing solution for event detection [14,15], with Mel Frequency Cepstrum Coefficient (MFCC) being one of the most mature acoustic feature extraction methods for capturing the characteristic information of transient sound events [16,17,18,19]. Meanwhile, deep learning, especially lightweight convolutional neural networks (CNN), has realized high-precision classification of acoustic features with low computational overhead [20,21,22], laying a foundation for the on-site identification of impact events [23,24,25,26]. Regarding the embedded porting of intelligent algorithms [27], existing research has successfully ported neural networks to embedded platforms through lightweight network architecture design and hardware platform adaptation [28,29,30,31,32].

To tackle the aforementioned issues, this study presents a synchronous triggering method for impact sources and seismographs utilizing non-contact audio detection. A hardware system is constructed around the STM32F4 microcontroller. On the software side, the MFCC algorithm is employed to extract 39-dimensional acoustic features [33]. Additionally, a lightweight CNN model is developed to identify hammer impact events accurately. Furthermore, we implement a synchronization time compensation mechanism to address the time discrepancies caused by processing delays in the equipment.

The subsequent structure of this article is arranged as follows to systematically elaborate on the proposed synchronous triggering method. Section 2 focuses on the extraction of sound signal features at the moment of artificial seismic source impact, detailing the principle and specific implementation steps of the MFCC algorithm in extracting 39-dimensional acoustic features. Section 3 introduces the construction of the lightweight CNN model for audio classification, including the network structure design, the dataset construction process, and training and performance testing results. Section 4 describes the overall construction of the embedded system based on the STM32F4 microcontroller, as well as the field experimental testing conducted in three different environments, and analyzes the error compensation effect of the synchronization time compensation mechanism. Finally, Section 5 summarizes the full research work, discusses the existing limitations, and puts forward prospects for future optimization directions.

## 2. Sound Signal Feature Extraction at the Moment of Impact of the Artificial Seismic Source Based on MFCC

**Figure 2:**
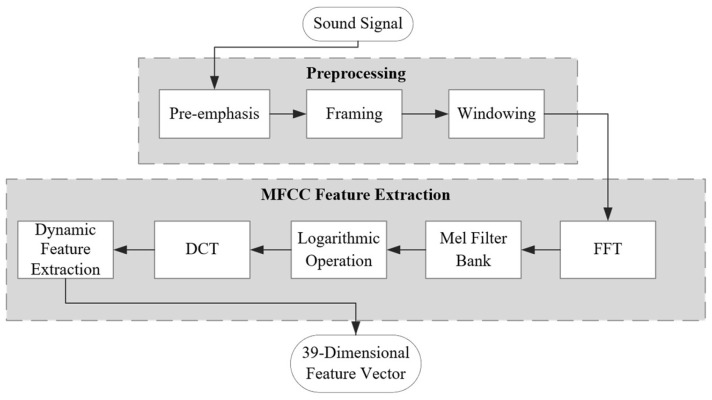
Flowchart of MFCC feature extraction.

The focus of this study is the sound signal produced at the moment of impact when an artificial seismic source interacts with an interface under a complex noise environment. This noisy environment typically consists of a construction site with active machinery and a tunnel section undergoing development. Effectively extracting hidden information from the original data using feature extraction algorithms in such settings is crucial for the subsequent success of classification algorithms. Given that the sound generated at the moment of impact differs significantly from other environmental noises in human perception, and that the signal characteristics are primarily concentrated in the mid-low frequency range, this study utilizes Mel Frequency Cepstrum Coefficient (MFCC) as the feature extraction method. MFCC processes the sound signal through a Mel filter bank to mimic the human ear’s sensitivity to varying frequencies of sound waves. The process involves several steps, including preprocessing, Fast Fourier Transform (FFT), application of the Mel filter bank, logarithmic compression, and Discrete Cosine Transform (DCT), ultimately yielding a 39-dimensional audio feature vector [34]. The feature extraction process, consisting of those 39 dimensions, is illustrated in Figure 2.

Preprocessing involves several steps, including pre-emphasis, framing, and windowing, among others. This phase addresses the issue of high-frequency attenuation through pre-emphasis, which helps to balance the high and low frequency spectrum, thus preventing numerical issues that may arise during subsequent fast Fourier transform operations. Following this, the signal is framed and windowed to create smooth, short-time frame signals, with the Hamming window employed to mitigate spectrum leakage [35]. The formula for pre-emphasis is

(1) $$y\left(n\right)=x\left(n\right)-\alpha x\left(n-1\right)$$

The term y(n) refers to the audio data after pre-emphasis, and x(n) refers to the original audio data. α represents the pre-emphasis coefficient, which typically ranges from 0.95 to 1 [36,37]. In this study, the value is taken as 0.97. The expression for the Hamming window function is

(2) $$\omega \left(n\right)=\beta -\left(1-\beta \right)cos\left(\frac{2\pi n}{N-1}\right)$$

The term ω(n) refers to the weighted window function; β refers to the window function coefficient, which is taken as $\beta =\frac{25}{46}$; n refers to the index representing the sampling points of the window function, 0≤n<N−1; N refers to the window length.

Feature extraction at the moment of impact should emulate the structure of the human ear to capture the sound signal effectively. The human ear exhibits varying sensitivities to different frequencies. To align more closely with this auditory mechanism, it is necessary to redefine the Mel frequency beyond the standard frequency range. The relationship between Mel frequency and frequency is logarithmic, which better approximates the human ear’s sensitivity to low-frequency sound signals and its insensitivity to high-frequency sound signals. The formula that defines the correspondence between Mel frequency and frequency is

(3) $$mel\left(f\right)=1125ln\left(1+\frac{f}{700}\right)$$

In order to more accurately mimic the nonlinear perception of sound by the human ear and to enhance its ability to distinguish impact moments, a Mel filter is employed to process the spectrum following the Mel frequency transformation. This approach divides the sound signal into nonlinear blocks at the Mel frequency, allowing for a more refined extraction of information [38]. The number of Mel filters typically ranges from 20 to 40. Related studies in the field of speech recognition have found that the specific number of filters has no significant impact on feature extraction and recognition accuracy [39]. To strike a balance between computational efficiency and feature extraction accuracy, this study adopts 26 as the standard number. The calculation formula for the Mel filter is as follows:

(4) $$H_{m}(k)=\left\{\begin{array}{l} \;\;\;\;\;\;\;\;\;\;\;\;\;\;0,k=f(m-1)\\ \frac{k-f(m-1)}{f(m)-f(m-1)},f(m-1)<k<f(m)\\ \;\;\;\;\;\;\;\;\;\;\;\;\;\;1,k=f(m)\\ \frac{f(m+1)-k}{f(m+1)-f(m)-f(m)},f(m)<k<f(m+1)\\ \;\;\;\;\;\;\;\;\;\;\;\;\;\;0,k=f(m+1) \end{array}\right.$$

The term $H_{m}\left(k\right)$ refers to the filter weight at the m-th Mel filter in k-th frequency point; f(m) refers to the filter weight at the m-th Center frequency index of the Mel filter; k refers to the frequency point index in the frequency domain, m refers to the index of the Mel filter, namely represents the m-th Mel filter.

The cepstrum operation involves separating the signal by first applying a logarithmic transformation to the Fourier transform spectrum, followed by an inverse Fourier transform. Compared to the Discrete Fourier Transform (DFT), the Discrete Cosine Transform (DCT) offers superior energy concentration in the frequency domain. Consequently, this study utilizes logarithmic operations on the signal and employs DCT, rather than DFT, to decorrelate the filter bank coefficients and extract Mel-frequency cepstral coefficients (MFCC) features [40]. The formula for the cepstrum operation is as follows:

(5) $$mfcc\left(i,n\right)=\sum_{m=0}^{N-1}log\left[H_{m}\left(i,m\right)\right]\cos\left(\frac{\pi n\left(2m-0.5\right)}{2M}\right),n=1,2,\ldots ,L$$

The term $H_{m}$ refers to the Mel filter array matrix obtained after Mel filtering; M refers to the number of triangular filters, which is 26 in this study; i refers to the i-th frame data, n represents the n-th column of i-th frame. *L* refers to the order of the MFCCs. The widely used range for this order is 13 to 42 [41]. In the field of speech recognition research, 13 is well-known as the optimum number [42]. Moreover, according to the characteristics of DCT, the first 13 coefficients retain the majority of the signal’s energy. Therefore, L is taken as 13 in this study.

The energy of the sound signal changes significantly at the moment of impact. Therefore, in addition to the static information conveyed by the signal, feature extraction must also capture dynamic information through first-order and second-order difference operations. Together, these elements—along with the static information—constitute a comprehensive 39-dimensional feature vector. The formulas for the first-order and second-order difference operations are as follows:

(6) $$dmfcc\left(i,j\right)=2mfcc\left(i+2,j\right)+mfcc\left(i+1,j\right)-mfcc\left(i-1,j\right)-2mfcc\left(i-2,j\right)$$

(7) $$ddmfcc\left(i,j\right)=2dmfcc\left(i+2,j\right)+dmfcc\left(i+1,j\right)-dmfcc\left(i-1,j\right)-2dmfcc\left(i-2,j\right)$$

## 3. Audio Classification Based on Neural Networks

### 3.1. Constructing a Neural Network

After generating the features of the sound signal, a classification algorithm is required to categorize these features, which exhibit various characteristics. Given the large volume of sound signal feature data, this study proposes a compact neural network designed to classify these features, effectively filtering impact moments from the microphone input stream. The Convolutional Neural Network (CNN), a deep learning architecture inspired by biological visual processes, enables autonomous learning of data features through multi-layer convolutional operations.

**Figure 3:**
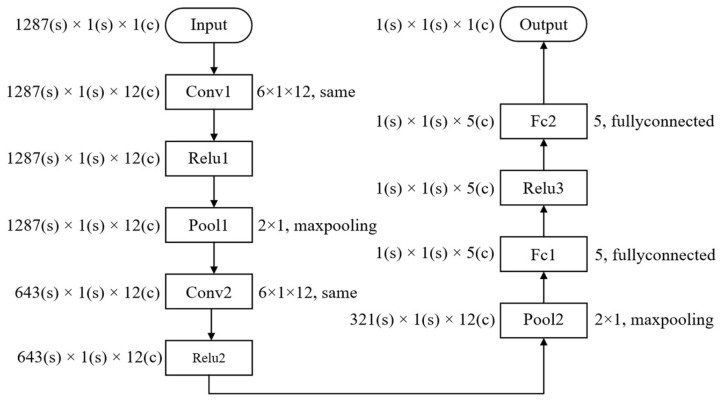
Neural network structure diagram.

Since synchronous triggering devices demand high-speed recognition of impact moments, the CNN model developed in this study is optimized to ensure high classification accuracy while also maintaining simplicity to enhance computational efficiency, paving the way for future embedding into systems. Therefore, a one-dimensional convolutional neural network (1D CNN), optimized for sequence models, is employed instead of the traditional two-dimensional CNN typically used for image processing. The input data, originally structured as (33, 39), is flattened into a 1D sequence of (1, 1287) before being fed into the CNN. Regarding the convolutional layer design, a 6×1×12 kernel with a stride of 4 is utilized. A receptive field of length 6, combined with this stride, aims to precisely extract the short-term transient energy pulses of the hammer impact, preventing larger kernels from introducing background noise and smaller ones from losing waveform information. Simultaneously, the 12 output channels strike an optimal balance between feature extraction capability and computational complexity reduction. For the fully connected layers, the model adopts a design with only five neurons, creating an information bottleneck at the end of the network. This forces the model to filter out redundant information during dimensionality reduction and retain only the core features essential for the binary classification task [43]. This design strictly limits the total number of learnable parameters to 20.2 k. The specific network structure is shown in Figure 3.

### 3.2. Neural Network Training and Performance Testing

#### 3.2.1. Dataset Construction

In this study, 86.67% of the training data is derived from laboratory sources, while the remaining 13.33% consists of data synthesized through mixing the original recordings. The research focuses on collecting audio data related to the energy release process in a simulated construction site environment, capturing both natural noise and distant construction sounds. A total of 1300 valid audio segments were gathered, comprising 410 segments of hammer impact and 890 segments of non-hammer impact. All samples were uniformly truncated or padded to a length of 512 ms. To simulate strong noise interference scenarios commonly encountered in real engineering projects, 8 hammer impact audio segments and 12 non-hammer impact segments were randomly selected from the original dataset. These were mixed with typical extraneous noises from construction environments, such as sounds from construction elevators and machinery, at a volume ratio of 4:1, resulting in 200 sets of processed audio files (80 hammer impact segments and 120 non-hammer impact segments). The training and test sets were then created from this dataset at a 7:3 ratio, finalizing the preparation of neural network training and test sets. During the data feature extraction stage, all audio samples were uniformly normalized using the min-max method before feature extraction. The extracted features were subsequently reformatted to the shape of (33, 39), and the feature extraction data was resized to (1, 1287) to align with the neural network input requirements.

Regarding the training speed of the neural network, since the training dataset involved in this study is small and the network structure is relatively simple, we do not place too much emphasis on the convergence speed and training time of the network. Therefore, the hyperparameters in the training process are set as shown in Table 1:

All experiments in this study were conducted on the MATLAB R2020a platform using the Neural Network Toolbox to build, train, and validate the neural networks.

#### 3.2.2. Analysis of Neural Network Training Results

**Figure 4:**
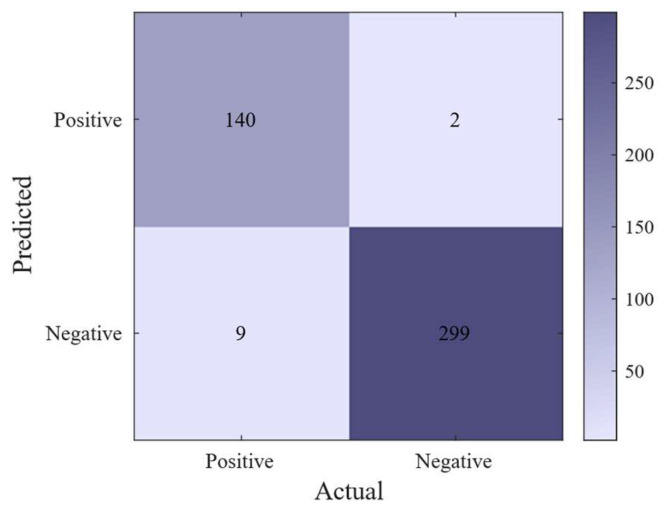
Neural network confusion matrix.

**Figure 5:**
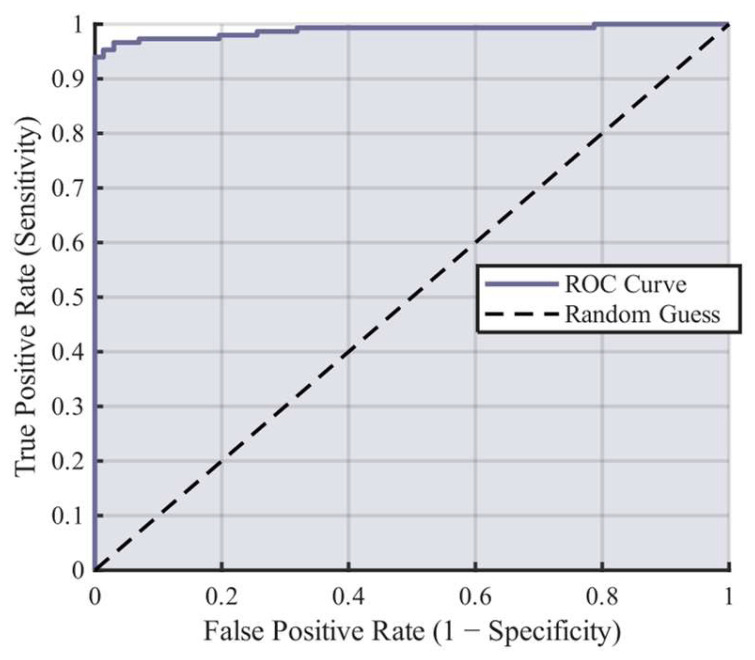
Neural network ROC curve.

The network achieved an RMSE of 0.15635 and an MAE of 0.0244 during training, indicating exceptional training performance. On the test set, the network achieved an accuracy of 97.56%, a precision of 93.96%, a misclassification rate of 2.44%, and an F1 score of 96.22% for classifying hammering sounds. The confusion matrix and ROC curve with the AUC of 0.988 are presented in Figure 4 and Figure 5. Notably, because both the F1 score and the ROC curve are robust evaluation metrics that are insensitive to class distribution, these high values strongly confirm that the model’s performance is not severely affected by the mildly imbalanced dataset. It proves that the network genuinely captures the core transient features of hammer impacts rather than merely biasing toward the majority background noise class.

Given its lightweight design, this study employs the X-CUBE-AI extension package to accomplish the porting and deployment of the neural network on the STM32F407 microcontroller. The memory occupation parameters after optimization are presented in Table 2. These results demonstrate that the network has excellent performance in accurately classifying hammer sounds while maintaining a streamlined structure.

## 4. Embedded System Construction and Experimental Testing

### 4.1. Embedded System Construction

**Figure 6:**
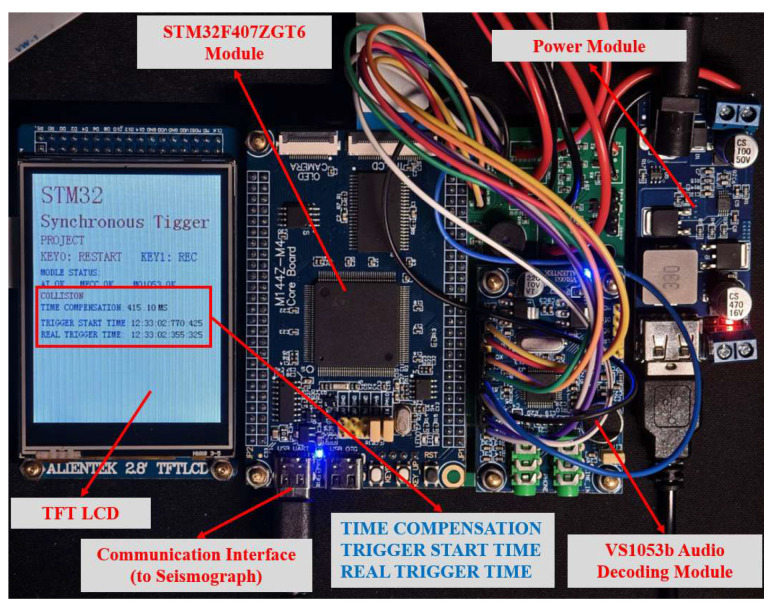
Hardware of the synchronous trigger.

To address the requirements of miniaturization, high computational capability, and multifunctionality in the embedded system explored in this study, this section outlines the selection of core hardware and circuit design. The STM32F407ZGT6 (STMicroelectronics, Geneva, Switzerland) module was chosen as the microcontroller unit (MCU), and the X-CUBE-AI from the STM32Cube (Version:6.14.1). AI extension package was utilized to integrate the neural network. Alongside the main control unit, additional components such as a TF card module, external SRAM, an LCD, and various peripherals (including LEDs, buzzers, and buttons) were selected to facilitate the storage and interaction capabilities of the triggering hardware. Moreover, the VS1053b audio decoding module was chosen for audio acquisition, which not only supports WAV storage but also meets the miniaturization demands of embedded systems. This module can be further expanded to function as an audio decoder, serving as a sample acquisition tool. The hardware of the synchronous trigger is shown in Figure 6.

**Figure 7:**
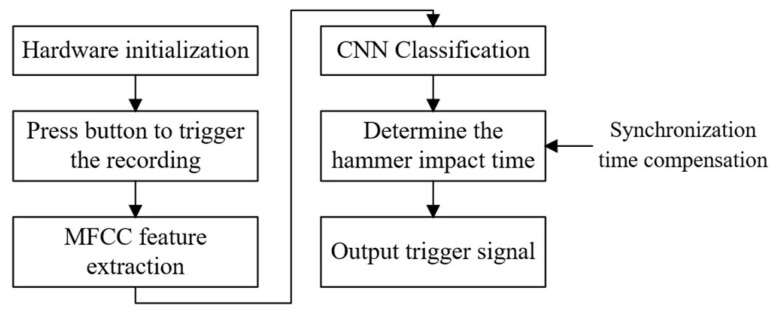
Workflow of the embedded synchronous triggering program.

This study ultimately uses the STM32F407 as the core, connecting VS1053b, TF card, TFT LCD, and other modules to build a complete synchronous triggering hardware system. Once the system detects the impact moment, it immediately outputs one rising-edge signal and one falling-edge signal through an aviation connector to synchronize with the seismograph. The signal parameters are presented in Table 3. The overall workflow of the system is illustrated in Figure 7.

To further minimize the impact of MCU performance on impact moment detection, this study introduces a synchronization time compensation scheme to reduce detection errors. When the hammer impact moment is detected, two time intervals arise that significantly interfere with the real-time performance of the synchronization signal. The first interval pertains to the calculation and determination time of the embedded system porting algorithm, which can be accurately measured by controlling the timer’s activation and deactivation, allowing for precise compensation. The second interval is the uncertain time between the sample acquisition and recording times and the specific moment of hammer-impact energy release. For compensation purposes, this study initially designates the midpoint of this interval as the reference time.

### 4.2. Experimental Testing of Synchronous Trigger Device

To replicate working conditions across different environments, this study conducted field tests in three distinct settings: an open space, a construction site, and a mining tunnel. Each environment underwent testing 100 times, culminating in a total of 300 experiments. The key indicators assessed were the accuracy of the synchronous trigger system and the error range of the output synchronous trigger signal. The experimental process consisted of two main components: data acquisition and manual comparison. Initially, an artificial earthquake was simulated using an impact source. Upon detecting an impact event, the synchronous trigger system automatically saved and numbered the corresponding audio file at the moment of trigger and recorded the specific time elapsed since the start of the audio recording of the output synchronous signal. Following data acquisition, it was possible to assess on-site whether the synchronous trigger system had experienced a false trigger or had failed to trigger at all.

If the synchronous triggering system outputs a trigger signal within the correct time frame, an automated program analyzes the time-domain energy distribution of the corresponding audio file and extracts the highest energy value as the marker for the actual impact moment. The discrepancy between the actual impact moment and the output timing of the synchronous trigger signal represented the error. Due to the extremely high sound energy produced by the impact source during the artificial earthquake, the likelihood of other sounds exhibiting the same high energy within a very short timeframe is minimal. Thus, the actual impact moment determined by this method is considered reliable.

**Figure 8:**
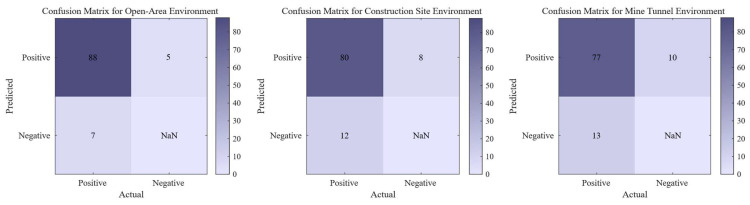
Confusion matrix of the synchronous triggering system test results in different environments.

The confusion matrix of the synchronous triggering system test results in different environments is shown in Figure 8.

**Figure 9:**
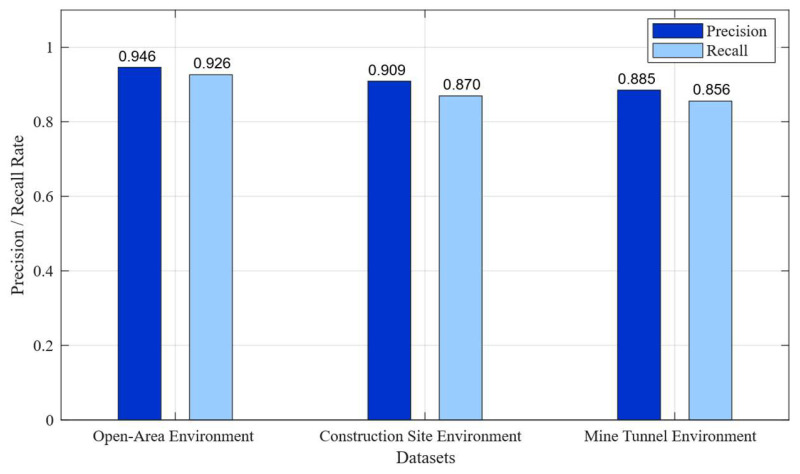
Precision and recall of the synchronous triggering system in different environments.

The precision and recall of the synchronous triggering system in different environments are shown in Figure 9.

**Figure 10:**
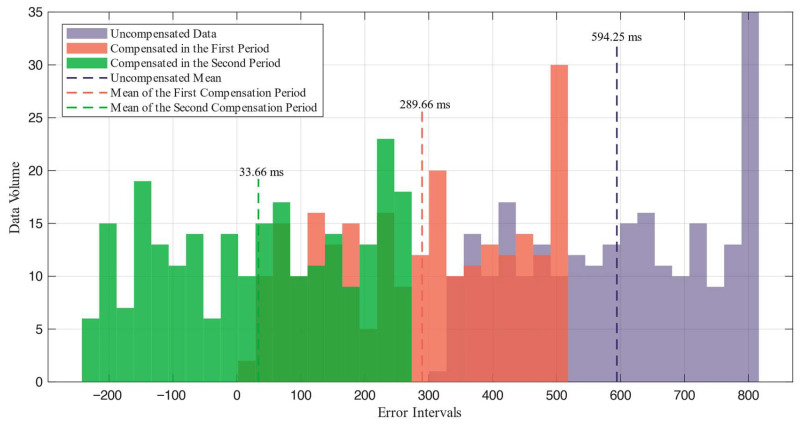
Comparison of error distributions after multiple compensation.

For the 245 data sets that produced correct judgments, the specific error value was calculated using the method outlined in the experimental design section. Additionally, the error distribution was determined after no time compensation, after the first time period compensation, and after the second time period compensation, as visually compared in Figure 10. The mean, median, and standard deviation for each data set are shown in Table 4.

According to the experimental data, after removing the initial time period—which accounts for the execution time of the microcontroller program—the time error is reduced to 48.74% of the uncompensated data. Further analysis shows that after subtracting a second compensation period from the first, the time error decreases to just 5.7% of the uncompensated data. This demonstrates that the implemented compensation strategy significantly enhances system accuracy. Given the relatively uniform probability of impact event generation during the recording period, the errors tend to be evenly distributed across a specific time range. Consequently, applying a fixed time of 250 ms for the second stage compensation during the experiment does not effectively reduce the standard deviation of the errors post-compensation, indicating the need for an alternative compensation strategy. If the code designed to detect the highest energy moment as the impact moment in the experiment is integrated into the microcontroller, the program can directly identify the peak energy value of the corresponding audio, using that moment for final compensation. By calculating the duration from this point to the end of the audio based on the sampling frequency and adding it to the algorithm’s running time, we can establish the final synchronization compensation time. The final compensation formula is as follows:

(8) $$t_{c}=\frac{N-1-n}{f_{S}}+t_{cul},0\leq n\leq N-1$$

**Figure 11:**
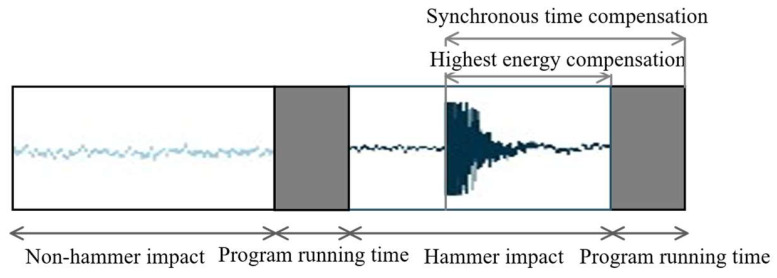
Schematic diagram of improved synchronization time compensation and system timing.

The term n represents the position of the point from the initial to the highest energy peak. The notation N−1 indicates the total number of sampling points, $f_{S}$ denotes the sampling frequency, $t_{c}$ refers to the synchronization compensation time, and $t_{cul}$ represents the algorithm calculation time. The enhanced synchronization time compensation and the associated system timing diagram are illustrated in Figure 11. The range of time error in the improved synchronization compensation is solely determined by the gap period caused by the sampling interval of the audio samples. This approach significantly reduces both the standard deviation and the average of the error. After synchronization compensation, the remaining time error is solely attributed to the 8000 Hz sampling frequency. Because the initial highest energy point may arrive between two sampling points, the synchronization compensation algorithm can only localize the true impact moment within the ±125 µs sampling interval. Consequently, the absolute time error of the hammer impact moment falls strictly within the range of [−125 µs, 125 µs]. This level of accuracy successfully satisfies the design requirements for the synchronization trigger.

## 5. Conclusions

To address the synchronization error between the impact source and the seismograph, which arises from the uncertainty in the hammer’s acceleration time, this study investigates a non-contact synchronization triggering technique. The system developed employs MFCC technology to extract 39-dimensional features from 512 ms of audio. A lightweight convolutional neural network has been designed to balance classification accuracy with embedded computing power requirements and is trained using manually collected audio data related to impact experiments. On the test set, the system achieves a classification accuracy of 97.56%, a precision of 93.96%, and a misjudgment rate of 2.44%. Furthermore, a dynamic time compensation scheme was introduced to mitigate the impact of composite errors on accuracy. The system underwent 300 field tests across three environments: open ground, construction sites, and mining tunnels, with accuracy rates of 94.6%, 90.9%, and 88.5%, respectively. Through the implementation of dynamic time compensation, the synchronization error was reduced from 594.25 ms to 33.66 ms. Moreover, additional refinements based on the highest audio energy points allowed the absolute time error to be controlled within the range of [−125 µs, 125 µs], thereby meeting the design requirements for the synchronization trigger and successfully realizing the construction of a non-contact synchronization trigger system. It can serve as auxiliary equipment for artificial seismic sources. Owing to its non-contact design, the system is immune to the intense electromagnetic pulses and mechanical vibration interference generated by impact sources. Compared with piezoelectric vibration sensors or photoelectric switches, it offers an extended service life and superior system stability. This system is particularly well-suited for engineering projects in explosive-restricted urban areas or confined tunnel environments characterized by significant construction noise.

This study presents three areas for potential optimization:

(a) Insufficient Scene Coverage in the Training Dataset: The current dataset lacks a comprehensive collection and integration of tunnel noise in complex environments, resulting in a limited dataset size. This limitation significantly hampers the model’s performance in real tunnel scenarios.
(b) Handling Extreme Class Imbalance: Although the natural data ratio used in this study did not severely impact the model’s performance, real-world extreme noisy environments might present a much more skewed ratio. Future work will explore class balancing strategies, such as class weighting or focal loss mechanisms, to further enhance the model’s robustness under heavily imbalanced conditions.
(c) Inadequate Performance of Audio Acquisition Hardware: The microphone’s signal-to-noise ratio and frequency response are not ideally suited for the low-frequency characteristics of the hammer impact signal. Additionally, the maximum sound pressure level is insufficient to effectively capture instantaneously strong sound signals, leading to issues such as weak signals being obscured and strong signals being clipped and distorted.
(d) Performance Bottlenecks in the Embedded Platform: The constraints of the existing embedded platform limit advancements in system processing efficiency and model complexity. Selecting a higher-performance microprocessor unit (MPU) could significantly enhance the performance of the synchronization trigger.

This research offers technical support for the practical application of artificial seismic sources, aiming to reduce reliance on explosive sources while emphasizing safety and environmental preservation. Future work could focus on further optimizing the model’s robustness against extreme noise to accommodate more complex survey scenarios. Additionally, employing a more powerful microcontroller could help decrease program execution time and alleviate the pressure for time compensation.

## Figures and Tables

**Table 1:** Neural network training parameters.

Optimization Function	Maximum Number of Training Rounds	Batch Size	Initial Learning Rate	Gradient Threshold
Adam	40	16	0.005	1

**Table 2:** Memory occupation parameters of the optimized neural network.

Complexity (MACC)	Used Flash (KB)	Used RAM (KB)
715,129	79.15	35.68

**Table 3:** Electrical parameters of the output trigger signals.

Type	Pulse Width	Output Voltage	Maximum Allowable Current
Pulse	500 ms	12 V	500 mA

**Table 4:** Statistics of errors after multiple compensations.

Uncompensated Data			First Compensated Time Period			Second Compensated Time Period		
Mean	Median	Standard Deviation	Mean	Median	Standard Deviation	Mean	Median	Standard Deviation
594.25	604.15	150.25	289.66	299.25	150.26	33.66	43.25	150.27

## Data Availability

The datasets presented in this article are not readily available due to technical limitations.
